# Acceptability of midazolam and melatonin as premedications for anxious children undergoing general anaesthesia: a qualitative interview study with children, caregivers and health professionals participating in the MAGIC trial

**DOI:** 10.1186/s13063-024-08611-x

**Published:** 2024-12-05

**Authors:** Jennifer Kettle, Robert Bolt, Chris Deery, Diana Papaioannou, Helen Rodd, Marie C. Hyslop, Nikki Totton, Zoe Marshman

**Affiliations:** 1 School of Clinical Dentistry, University of Sheffield, Sheffield, UK; 2 Sheffield Clinical Trials Research Unit, School of Medicine and Population Health, University of Sheffield, Sheffield, UK; 3 Population Health, School of Medicine and Population Health, University of Sheffield, Sheffield, UK

**Keywords:** RCTs, Qualitative, Paediatric anaesthesia, Paediatric anxiety

## Abstract

**Background:**

The acceptability of a children’s premedication, prior to general anaesthesia (GA), is fundamental to ensuring positive clinical- and patient-reported outcomes. Midazolam, the current standard premedication, is known to have an unfavourable side-effects profile and presents a degree of risk which is accepted due to a need for compliance. Melatonin is a functionally diverse hormone with anxiolytic properties that offer potential benefits over midazolam. Little is currently known about how patients and health professionals view these two different premedications. This research aimed to explore the acceptability of midazolam and melatonin as premedications for anxious children undergoing GA, from the perspective of children, caregivers and health professionals involved in the Melatonin for Anxiety prior to General Anaesthesia In Children (MAGIC) trial.

**Methods:**

Participants were children recruited to the MAGIC trial, their caregivers and health professionals involved in recruitment to the trial. In total, 37 participants (23 health professionals, 10 caregivers and 4 children) took part in semi-structured interviews relating to the MAGIC trial and acceptability of premedications. Interviews were carried out face-to-face, by telephone or online by a trained qualitative researcher. Interviews were transcribed verbatim and analysed using a framework approach.

**Results:**

The acceptability of midazolam and melatonin is related to six main factors: effectiveness as premedication prior to GA; administration of premedication; experience of recovery; prior experiences of premedication; associations and evidence; and range of options for managing anxiety. Interviews highlighted the trade-offs involved and the relevance of the wider context in which premedications are provided. Barriers and facilitators were identified on the acceptability of premedications more generally.

**Conclusions:**

Future clinical trials evaluating the effectiveness of premedications in children prior to general anaesthesia need to consider that premedication choice is multifactorial. The MAGIC study found that melatonin was less effective at reducing anxiety (pre-operative distress) when compared with the standard of care, midazolam. However, there remains a need for a premedication with a better side effects profile to midazolam. While children, caregivers and health professionals are open to alternatives to midazolam, this is likely to vary by subgroup and will involve trade-offs in terms of benefits.

**Trial registration:**

ISCRCTN ISRCTN18296119. Registered on 10/01/2019.

## Background

Preparing for a surgical or medical intervention under general anaesthesia (GA) can, understandably, be a stressful event for a child and their family. Anxiety is common in paediatric patients, with up to 50% of children displaying distress at the point of anaesthetic induction [1]. To help children accept the necessary procedures, and to make the whole experience as positive as possible, anaesthetists may sometimes advise the use of a premedication. Although a number of different premedication agents are currently used for paediatric patients, none are considered ideal as they all have limitations and potential side effects [2]. In the UK, midazolam is widely adopted as the ‘standard’ premedication for anxious children [3]. However, it is recognised as having an unfavourable side-effects profile, notably, causing some children to experience postoperative emergence delirium (a distressing state where children may cry inconsolably, kick, scream and appear uncooperative) [4]. Midazolam also has a bitter taste, with the risk that children may refuse to swallow it; and if given nasally it reportedly causes mucosal irritation [5].

In view of these acknowledged limitations, interest has turned to the use of melatonin as an alternative premedication, for both children and adults. Melatonin is a natural sleep-promoting neurohormone that has promising potential as a pre-operative anxiolytic. Purported benefits of melatonin over midazolam include more acceptable taste, ambulant rather than bed transfer to theatre, faster recovery times, improved postoperative analgesia, reduced postoperative sedation, and a lower incidence of subsequent sleep disturbances [6, 7]. A recent randomised double-blind placebo-controlled study of 3–8-year-old children evaluated the incidence of emergence delirium and concluded that melatonin significantly reduced this occurrence, compared to midazolam or a placebo [8]. A systematic review of pre-operative melatonin use in children has also highlighted its excellent safety profile although the authors could not confirm whether it was inferior to comparator drugs in terms of effectiveness as a premedication [9].

It is worth noting that the majority of these studies have employed biomedical measures to compare outcomes, such as recording physiological parameters or using established scales to record a child’s behavioural responses or degree of sedation [2]. The experiences and views of children, their families/carers and healthcare professionals appear to have been largely overlooked. Although there is some mention of ‘satisfaction’ in a few studies, sparse detail is provided as to how this was actually measured. For example, a comparison of the effectiveness of melatonin versus midazolam as a premedication for intravenous (IV) sedation in paediatric dental patients concluded that midazolam was the superior agent and that there was higher operator and parental satisfaction, but did not explain how and why this was determined [10]. There have been no previous qualitative research comparing views on the acceptability of midazolam and melatonin as children’s premedications. Therefore, there is little understanding on how premedications are experienced by children and carers, as well as a lack of research on the decision-making processes of anaesthetists regarding choice of paediatric premedication.

The Melatonin for Anxiety prior to General Anaesthesia In Children (MAGIC) randomised controlled trial (RCT) was a UK-based double-blinded, multicentre parallel randomised controlled non-inferiority trial. The trial was designed with the primary objective of comparing the effectiveness of melatonin and midazolam as pre-medications for anxious children prior to GA, using the modified Yale Preoperative Anxiety Scale (mYPAS) at four time measurement points (holding, start of transfer to theatre, entry into the anaesthetic room and on the administration of anaesthesia). The hypothesis was ‘melatonin is not inferior to midazolam in reducing anxiety in children pre-GA, with fewer side effects’. Secondary outcomes included comparisons of safety outcomes, efficacy outcomes, cost-effectiveness and acceptability [11]. Qualitative research can be used to explore the acceptability of treatment options within a randomised controlled trial (RCT) [8]. Interviews with health professionals, patients and caregivers offer multiple perspectives on the acceptability of a healthcare intervention.

The MAGIC trial closed early due to recruitment futility. However, the pre-defined Intention-To-Treat and Per-Protocol analyses found melatonin is inferior to midazolam in reducing anxiety; with the difference in reduction of anxiety being both clinically meaningful and statistically significant Non-inferiority trials test whether a new treatment is as good as i.e. no worse than an existing treatment, whilst also offering additional benefits e.g. being cheaper or having fewer side effects. Whilst melatonin appears less effective than midazolam for this indication, there remains a need to identify an effective premedication with a better side effects profile. However, attributes of alternative premedications to midazolam, e.g. amnesic effect, may make a new proposed drug more or less favourable with children, caregivers or clinicians, or that it may be acceptable for some subgroups and not others. Exploring the acceptability of premedications can allow understanding of the multiple factors influencing premedication choice, which may be important to future non-inferiority trials in this area.

This paper reports on the acceptability of midazolam and melatonin from the perspective of children, caregivers and health professionals, and barriers and facilitators to premedication acceptability.

## Methods

### Study design

During MAGIC’s internal pilot, a qualitative study was conducted with children, caregivers and stakeholders (including those who contributed to the trial design, anaesthetists and research nurses), children and caregivers, with the aim to identifying barriers and enablers to MAGIC trial recruitment (data on barriers and facilitators to recruitment are reported elsewhere [12]). Following the start of recruitment, further interviews were conducted to explore the acceptability of midazolam and melatonin. Qualitative research was used to bring together in-depth accounts from multiple perspectives. Methodologically, the qualitative study was based on ‘subtle realism’ (i.e. an external reality exists outside of people’s interpretations, but can only be accessed through these interpretations, not directly) [13]. Semi-structured interviews were used in order to focus discussions on the topic of premedication acceptability, while allowing the interviews to explore related topics.

### Recruitment

UK trial sites were purposively sampled on the basis of type of setting, region and success with recruitment (pilot phase: eight sites; main phase: thirteen sites). Stakeholders were approached by email and invited to take part in an interview. Stakeholders approached at each site were principal investigators (PIs) and research nurses. They were also asked to provide details of other health professionals, particularly anaesthetists responsible for recruiting to MAGIC, and nurses working in surgical day units with anxious children. In the pilot phase, one PI declined to take part in the qualitative study due to time constraints and one did not respond to multiple attempts at contact; the site subsequently stopped recruiting patients. In the main phase, five PIs did not respond. Of the eight PIs that responded, one was not available to interview. One responding PI recommended interviewing another consultant paediatric anaesthetist colleague, who was subsequently recruited instead of the PI. Seven interviews were arranged. Research nurses from four sites were approached. One did not respond. Of the three research nurses who responded, one was new to MAGIC and did not want to take part. Two interviews were arranged.

The trial management team provided details of caregivers who had agreed to be contacted by the research team about the qualitative study. Telephone calls were made to all these caregivers who were asked if they were willing for their child to take part in this follow-on qualitative enquiry. In the pilot study, caregivers from four families were interviewed; children from two families were interviewed. In the main study, two caregivers did not respond, and two responded but were not available for an interview. Caregivers from three families were interviewed; children from two families were interviewed.

### Ethical considerations

The qualitative study was approved by the North West – Liverpool Central Research Ethics Committee as part of the MAGIC trial (IRAS 228234). Prior to the interview, all adult participants were provided with an information sheet about the qualitative study and given the opportunity to ask questions. Age-appropriate information sheets were provided for children. Participants were reminded that the interviews were voluntary, and could be stopped at any time. All adult participants provided verbal informed consent at the time of the interview. Caregivers provided consent for children to take part, and children also provided verbal assent.

### Data collection

Data collection took place at two phases of the trial: for the pilot phase, between November 2019 and March 2020, and for the main phase, between October 2020 and January 2021. All interviews were conducted by the first author (JK). JK is female, has a PhD, is experienced in conducting qualitative research and is not a clinician. The interviewer was independent of the main trial team. The independence from the trial team and lack of clinical experience were disclosed at the start of the interviews. One anaesthetist and a research nurse were interviewed together. One pair of caregivers were interviewed together. Two groups of caregiver-child dyads were interviewed together. No non-participating individuals were present. Interviews with stakeholders during the pilot stage took place in person (in private offices/meeting rooms) or by telephone. In the main study, all interviews were remote and took place by telephone or by video conference (these interviews took place during the COVID-19 pandemic). Interviews with caregivers/children for the main study took place in person in people’s homes or by telephone. In the main study, all interviews took place by telephone.

Interviews with caregivers and children averaged 27 min and ranged from 10 to 38 min. Interviews with stakeholders averaged 65 min, and ranged from 34 to 105 min. Interviews were audio-recorded, transcribed verbatim by an external company or the researcher, and checked by the researcher to ensure data quality. Notes were made during the interviews to highlight significant points for further discussion. Two interviews (one caregiver-child dyad) failed to record; notes from the interviews were written up and analysed. Transcripts were not returned to stakeholders for checking. Two stakeholder participants from the pilot phase were involved in the trial and commented on the interpretation of findings (RB and CD). These two participants were known to JK prior to the interview; there were no existing relationships with any other participants.

Topic guides were developed for the main trial, drawing on findings of the pilot trial interviews, literature on the acceptability of interventions and discussion with the trial management group. The topic guides were developed by JK, and approved by another qualitative researcher with experience of conducting interviews as part of RCTs (ZM). The topic guides were not pilot tested. The topic guides were used flexibly to allow interviewees to discuss issues that were important to them.

Data collection in the pilot phase ended at a point where the intended sample (two to four stakeholders from each of five or six sites) had been recruited, in order to feedback findings to the trial team. Data collection from stakeholders in the main trial phase, and from children and caregivers at both stages, was limited to those willing to participate and was impacted by wider issues relating to the trial (see limitations, below). As the number of interviews was based on the response of stakeholders and caregivers, and this was impacted by the early closure of the trial, it is not possible to say that saturation was achieved.

### Analysis

Framework analysis was used as a pragmatic approach [14]. The analysis involved the following stages: identifying initial themes, labelling the data, sorting the data by theme and synthesising the data. Transcripts were read and re-read to achieve familiarity and coded by JK. Themes were identified and organised into a framework, including child anxiety prior to GA, non-pharmacological management of anxiety, acceptability of premedications, acceptability of midazolam and melatonin within MAGIC, and potential future use of melatonin. The initial framework was discussed by JK and ZM and revised, focusing on two main themes: acceptability of midazolam and melatonin, and barriers and facilitators to premedication acceptability. Fourteen sub-themes were identified. Data was synthesised using the finalised framework (see Table 1: Finalised acceptability framework). Data was managed using Microsoft Word and Microsoft Excel.

**Table 1:** Finalised acceptability framework

**Acceptability of midazolam and melatonin**	
Effectiveness prior to administration of GA	
Administration of premedication	
Experience of recovery from GA	
Prior experience of midazolam and melatonin	
Associations and evidence	
Range of options for premedication	
**Facilitators and barriers to premedication acceptability**	
Prior experience with individual child	
Child engagement with premedication	
Caregivers’ attitudes to premedication	
Health professionals’ attitudes to premedication	
Health professionals’ attitudes to evidence	
Health professionals’ skills	
Managing known risks	
Systems and processes for premedication	

## Results

Overall, 37 participants were recruited (23 health professionals, 10 caregivers and four children) (see Table 2 for participant characteristics). The health professional interviews comprised 11 anaesthetists, nine research nurses and research leads, and three dentists. Two anaesthetists were interviewed in both the pilot phase and the main phase.

**Table 2:** Participant details

Role in trial	Role (if health professional)	Phase of trial	Region (if health professional)
Health professional (23)	Anaesthetist (11)	Pilot phase (4) Main phase (5) Pilot phase and main phase (2)	North East England (2) North West England (1) South East England (1) Yorkshire and Humber (2) Scotland (5)
	Research Nurse/ Research Lead (9)	Pilot phase (7) Main phase (2)	North East England (2) Yorkshire and Humber (3) South East England (1) Scotland (3)
	Dentist (3)	Pilot phase (3)	North East England (1) Yorkshire and Humber (2)
Caregiver (10)		Pilot phase (6) Main phase (4)	
Child (4)		Pilot phase (2) Main phase (2)	
**Total participants: 37**			

The analytical framework was based on two themes; the acceptability of midazolam and melatonin, and the facilitators and barriers to premedication acceptability; and 14 subthemes (see Table 1: Finalised acceptability framework). Subthemes are illustrated with anonymised quotations. In the reference numbers, PS and MS denote the pilot study and main study respectively. C denotes child, P denotes parent (or other caregiver) and S denotes stakeholder.

### Themes

#### Acceptability of midazolam and melatonin

##### Effectiveness prior to administration of GA

Midazolam was described as generally effective in facilitating the administration of GA. Anaesthetists noted the benefits of midazolam for everyone involved in the administration of GA:

I thinkwhat something like a pre-op benzodiazepine does is just take the sting out of the whole equation and makes it much more, makes the experience much more pleasant for everyone including the child, the parents, the nurses and for us. (Anaesthetist, South East England, MSS3)

This was important when children were especially anxious due to being unprepared, or due to developmental disorders and learning disabilities. Giving a child a pleasant experience was particularly helpful if he or she was likely to need to return for further treatment, and thus the choice of premedication could be an important facilitator.

Midazolam has a short onset, allowing children to quickly transfer to theatre and can make induction easier for anaesthetists:

I think midazolam works in a different way and there’s more of a chance that the child will really be quite sleepy and, therefore, induction will be easier for them and, you know, with respect to the anaesthetist, they’re the ones who are putting the children to sleep. (Research nurse, Yorkshire and Humber, MSS6)

Anaesthetists may have different intentions depending on the individual child (such as specifically wanting a child to be sleepy rather than merely happy and compliant), which may affect the choice of premedication. However, anaesthetists also commented on variations in the effectiveness of midazolam. This included hyperactivity (particularly among children with autism spectrum disorder) or becoming aggressive (like an ‘angry drunk’ (Anaesthetist, Scotland)), and thus uncooperative. This could lead to a stressful experience, or a need to reschedule.

Anaesthetists who were part of MAGIC viewed melatonin as potentially effective, particularly for certain groups of children:

You can have a frank discussion with a child who’s anxious about having the surgery and melatonin might be a good option for them in terms of they’recompliant and engaging. Whereas there are children who are verging on terrified, I’m not sure that it’s a good option for them in terms of… because you may reduce their anxiety a little bit but their experience from that isn’t going to be a good one. (Anaesthetist, Scotland, MSS5)

Determining how melatonin would fit into current anaesthetic practice was felt to depend on the results of the MAGIC trial. Nevertheless, among anaesthetists more generally, there may be concerns about the effectiveness of melatonin: ‘I guess people’s perception of melatonin is that it is weak […] maybe the perception of it it’s less potent a drug or something, I don’t know’ (Anaesthetist, Scotland, PSS12).

Although staff involved in MAGIC were blinded, familiarity with midazolam meant staff were developing different views on the effectiveness of melatonin and the implications for children:

Our feeling during the study was that it probably wasn’t as effective as midazolam […] I think the kids who are coming with midazolam were, who had taken midazolam, more likely to be asleep and drowsy. Whereas the ones with melatonin will be calmer a little bit, I think. (Anaesthetist, Scotland, MSS5)

I think that was melatonin the way he behaved and it’s amazing, that was so good. (Research lead, North East England, PSS14—quoting a theatre nurse)

As discussed below, positive personal experiences are key to professional acceptability, and despite blinding, experiences within MAGIC may contribute to this.

Caregivers and children reported their experiences of premedications within MAGIC. Caregivers reported varied effects prior to surgery, including children becoming ‘giggly’ (Caregiver, PSP1), ‘drunk’ (Caregiver, MSP1) and ‘chilled’ (Caregiver, PSP6). In terms of anxiety, caregivers emphasised effectiveness, reporting that children were ‘calmed down’ (Caregiver, PSP4) or ‘out of it’ (Caregiver, PSS1). There were also examples of children being ‘worried’ (Caregiver, PSP3) when arriving in theatre, but not to the extent this was described as problematic. Based on this small sample, children and caregivers were positive about whichever premedication they had received. There was one exception: a caregiver and child interviewed as part of the main phase reported that the premedication had no effect, and there were ‘no benefits’ (Caregiver, MSP3) from taking part.

##### Administration of premedication

A particular issue with midazolam is the bitter taste, and palatability to children:

It tastes horrible. So, the oral preparation doesn’t taste very nice. It’s quite bitter I believe. (Anaesthetist, Scotland, MSS1)

This can become a clinical problem:

I haven’t seen that many spit it out but I’ve seen colleagues come and talk to me about the fact that we need to get a different drug or formulation because their child spat it out and wouldn’t have it. (Anaesthetist, South East England, MSS3)

However, it is important not to overstate this. While taste is an issue, spitting out a premedication can reflect anxiety rather than palatability:

I think the child that spits it out is, not always, but would be liable to spit out anything that I give them, unfortunately. […] Some kids just have the trust issue that you know, they simply won’t take it. (Anaesthetist, North West England, MSS4)

Participants reported being able to make midazolam more palatable by mixing it with juice, (see the ‘ Managing known risks’ section).

Children and caregivers’ reports of taste varied and included negative assessments (‘awful’ (Caregiver, PSP6), ‘disgusting’ (Child, PSC1) and ‘funny’ (Caregiver, PSP4)). In other instances, children had made no comment and caregivers stated there were no issues: ‘No, no, she just took the whole lot, dead calm, through a syringe’ (Caregiver, MSP1). Research nurses reported some negative comments on taste and instances of children spitting out some of the premedication. Overall, the interviews indicated this was not a significant problem, and emphasised children’s acceptance of whichever premedication they were given (even if not liking the taste).

#### Experience of recovery from GA

Children’s recovery from GA and surgery can be challenging for caregivers. It was reported some nurses working in surgical day units ‘hate midazolam’ because of the challenging aftereffects:

But it’s very distressing to see your child, you know, coming round and thrashing about and not wanting you […]It’s just not very nice. (Research nurse, Yorkshire and Humber, MSS6)

As this nurse explained, this can be a barrier to maintaining a ‘congenial atmosphere’ for other children and families. Melatonin was perceived as having the potential to avoid this, being ‘cleaner’ and an option that ‘doesn’t cause delirium and craziness in autistic children afterwards.’ (Anaesthetist, North East England, PSS1). It was reported that nurses working in surgical day units were open to the idea of melatonin, particularly if there were fewer side effects for children.

Anaesthetists reported that caregivers could occasionally be deterred from accepting a premedication due to risks of post-recovery side effects (including sleep disturbance and other behavioural problems such as bedwetting). Anaesthetists also noted prolonged sleepiness following midazolam, which could delay discharge. A delayed discharge could then cause problems for caregivers and anaesthetists:

The child comes at the centre of a family. So, if I suddenly decide to admit a child and mum has to stay, I need to know that mum has planned the childcare with the other kids […] Because otherwise, mum will be hugely stressed out and unable to focus on the care of her own child in hospital, and that would affect the anaesthetic. The child would pick up on mum’s stress. (Anaesthetist, North East England, MSS2)

However, it was also noted that caregivers can be willing to make trade-offs, and accept a child’s distressed behaviour or a delayed discharge in the context of successful procedure under GA;

Once the child has had their surgery, the parents areso grateful and so relieved that they’ve had their surgery, any behaviour on the part of their child is… they just accept. (Research nurse, Yorkshire and Humber, MSS6)

It doesn’t matter how long it takes her to wake up as long as she wakes up and comes home. (Caregiver, MSP2)

Within MAGIC, caregivers were generally positive about their experiences of children’s recovery. Children were described as initially ‘disoriented’ (Caregiver, PSP2), ‘groggy’ (Caregiver, PSP1) and ‘a bit upset’ (Caregiver, PSP3). On return home, caregivers described a lack of problems, with children being ‘normal’ (Caregiver, PSP6) and ‘in good form’ (Caregiver, PSP5). Various post-operative symptoms were described by caregivers/children, including vomiting, drowsiness and anger. These experiences were not presented as significant problems, or in any way unacceptable. As noted above, there was an exception (MSP3, MSC2), where the caregiver and child emphasised negative experiences of recovery following an ineffective premedication (the child felt sick, was unable to eat, and while sleepy, was not able to fall asleep).

The amnesic side-effect of midazolam can be perceived as an advantage. However, it can be problematic for children requiring multiple procedures, as they will not remember the benefits of taking midazolam:

The problem is though with midazolam that they forget that they were nice and relaxed, so it gives you amnesia, so then they basically are reliant on always getting the midazolam because they don’t remember that they were actually nice and calm. So if melatonin was able to calm people down but allow them to remember that they were nice and calm then maybe they wouldn’t always need to get a pre-med. (Anaesthetist, North East England, MSS8)

Due to the lack of this side effect, melatonin offers a potential longer-term benefit for this group of patients.

#### Prior experience of midazolam and melatonin

For caregivers, premedications are viewed through the lens of previous experience. Both melatonin and midazolam are more likely to be accepted where there is personal familiarity that is not actively negative (e.g. giving melatonin to another child to help with sleep, or prior experience with midazolam as a premedication). In prior experience, midazolam may have been preferable to anxious behaviour (screaming, hitting and kicking); one caregiver whose child had previously received midazolam as a premedication felt it had a ‘decent effect’ (Caregiver, MSP4). However, prior experience may also be challenging (the same caregiver reported aggressive post-operative behaviour at home). On the basis of prior experience in a different context, melatonin offers a potentially more acceptable alternative:

I think the idea of being able to use melatonin instead of drugs is amazing, to be honest. Obviously, it’s synthetic but it’s not - your body just processes it differently. I’ve got a child that has ADHD so I do know the… rough kind of melatonin effect. Yeah, no, it’s quite amazing just to know that it can be done that way, or could be. (Caregiver, MSP4)

Anaesthetists, commenting on their own experiences and those of theatre nurses, made a connection between familiarity and use:

Because midazolam has been so widely used and the nurses are very familiar with it, most clinicians are very familiar with it, we tend to use in a vast majority. (Anaesthetist, South East England, MSS3)

This could also apply to melatonin, where staff had experience in other contexts (such as helping children with autistic spectrum disorder fall asleep). Where prior experience with midazolam had been positive, it could be used exclusively:

I find midazolam works really so I haven’t used anything else. (Anaesthetist, North East England, MSS8)

In this case, the anaesthetist tended not to use clonidine as a premedication because of ‘lack of familiarity’, and this could similarly be an issue with melatonin. Other anaesthetists were actively looking for alternatives:

There are many disadvantages to midazolam. So, if melatonin did work, it would actually help an awful lot. (Anaesthetist, North East England, PSS1)

#### Associations and evidence

From anaesthetists’ perspective, midazolam and melatonin are both potentially acceptable to caregivers with positive associations. It was suggested caregivers would accept midazolam as the ‘normal’ treatment while also being willing to accept melatonin as ‘normal in the body’, with the potential for fewer side effects (Anaesthetist, Yorkshire and Humber, PSS7). Although interviews indicated a lack of comments or questions from caregivers about either option, health professionals did report some particular interest in melatonin as ‘a more natural substance’ (Anaesthetist, Scotland, PSS12). The positive associations with melatonin as ‘*natural*’ also emerged in interviews with caregivers and while midazolam is accepted, these associations may make melatonin more acceptable to caregivers.

However, some caregivers may choose not to take part in a trial due to a fear of the unknown: ‘they don’t want to go out of their comfort zone or feel like it’s, you know, it’s not proven treatment’ (Caregiver, PSP2). This can relate to the serious nature of the situation (i.e. surgery under GA) and the risk of a child continuing to display anxiety, so caregivers may reject a trial (and by implication melatonin) in order to get ‘the drug that works’ (Anaesthetist, Scotland, MSS7). When asked about a hypothetical scenario after MAGIC where melatonin has been shown to be effective, and anaesthetists are willing to offer it, it would lose its association with being unknown. There was no indication caregivers would see it as less acceptable in this situation.

Evidence from the trial could be important, if caregivers are given the choice:

I believe obviously if the new one does prove to be beneficial for most kids, that’s the one I’d choose. (Caregiver, PSP2)

When asked about hypothetical future procedures, caregivers expressed the view that they would be happy with either option, if supported by evidence of effectiveness:

I think whatever he had worked fine for him. But if there was a choice and they both worked, and they both didn’t have any major side effects, I think any of the medications would be fine. (Caregiver, PSP3)

Anaesthetists, and departments more generally, may also be ‘*open*’ to the idea of melatonin if evidence is available:

If it came out that melatonin is at least as good as midazolam, it’s something certainly which I would bring up in department meetings to discuss and see if we can consider ordering it. Obviously, the trial will hopefully give us a bit more information on side effects and the pluses and minuses of it. (Anaesthetist, South East England, MSS3)

With regard to disseminating the findings of MAGIC, anaesthetists emphasised the importance of sharing experience within a department. If anaesthetists within a department have the experience of success with melatonin (including in terms of patient acceptability), it could ‘promote itself’ (Anaesthetist, North East England, MSS2).

As discussed above, familiarity is a key benefit of midazolam, and it may be challenging for some anaesthetists to stop using something they feel works for children who are really anxious. In a context where anaesthetists ‘do things in different ways with the same results’, it may only be ‘sensible’ to adopt a new premedication if it ‘was massively more efficacious and had a lesser side effects profile’ (Anaesthetist, North East England, MSS8). It may also be that the practices of individual anaesthetists are influenced by views within their department, whether for or against introducing melatonin; departments may reach a consensus as to whether ‘we believe in it or we don’t’ (Anaesthetist, Scotland, MSS1).

#### Range of options for managing anxiety

Anaesthetists emphasised that a range of options for premedication is important:

I just think there’s no one right medicine but having more available and experience of using other drugs would be useful. (Anaesthetist, Scotland, MSS5)

Anaesthetists were clear that whatever the results of the MAGIC trial, melatonin would not replace the use of other premedications, and midazolam would still be used. Melatonin potentially offers another option, as one of the ‘tools in the armoury’ (Anaesthetist, North West England, MSS4) for particular cases, as do other premedications used outwith MAGIC.

Attitudes regarding the acceptability of midazolam and melatonin reflected this wider context. Other premedications discussed in these interviews included clonidine (which had the advantage of being tasteless) and dexmedetomidine. Clonidine was used when the anaesthetist was concerned about acceptability of midazolam, sometimes in combination:

What’s quite useful is, because of the different onset times, is to give a child some clonidine first. It doesn’t taste of anything small volume. And that will chill them out, and the dose might make them a little bit sleepy. It’s probably not enough to get them where you want them to be on its own. But by giving the clonidine first and then maybe waiting half an hour and then giving the midazolam, then they’re more accepting of the midazolam. (Anaesthetist, Scotland, MSS1)

Anaesthetists may prefer to use particular combinations of premedications that have previously been successful. However, combining drugs also risks additional side effects and delays discharge. Dexmedetomidine was also viewed positively as an alternative to midazolam, partly due to its acceptability (as delivered through an intranasal spray, it cannot be spat out). Nevertheless, it is more expensive, and cost is another consideration. Dexmedetomidine is not necessarily available in all departments and may also be clinically unfamiliar.

#### Facilitators and barriers to premedication acceptability

As well as specifically discussing midazolam and melatonin, interviews discussed the use of premedications more generally. This included facilitators and barriers that may be relevant when considering the acceptability of any premedication.

##### Prior experience with individual child

Anaesthetists emphasised the importance of being child-led and will consider a child’s previous experience of premedication:

Sometimes patients have paradoxical effects with the pre-meds where they become a bit disinhibited, you know, could become a little bit more agitated or bit more lairy, then obviously, that would influence and we’d probably try an alternative, rather than, you know, put them through the same thing. (Anaesthetist, North West England, MSS4)

Prior experience can be a barrier or a facilitator, depending on the child’s response to a particular premedication.

##### Degree of child engagement with premedication

Children’s willingness to accept premedication can be highly variable. Some older children are prepared to accept a premedication to help their anxiety. They recognise the benefits and will cooperate. It was not clear that melatonin would be any *more* acceptable than midazolam for this group, and acceptance/rejection could relate to premedications generally, rather than specific formulations:

I think sometimes when you discuss the options, more in that sort of direction, that they become either more convinced or they move away from the idea. Rather than different types of pre-med options that are given, you know. (Anaesthetist, North West England, MSS4)

Other children refuse to accept any premedication, are uncooperative and reject everything that is happening to them.

About a quarter of our children would have some kind of reluctance to engage with it and those are the children who refused to engage with anything. They just don’t want to be there. (Anaesthetist, South East England, MSS3)

These children may spit out anything and, due to their level of distress, may have to be discharged without undergoing GA, and require a rescheduled admission. This can reflect a level of anxiety and a lack of preparation and planning. Preparation emerged as a key issue, and a lack of preparation could make administering an anaesthetic more challenging; anaesthetists have ‘lost it from the start’ if preparation at home is ‘rubbish’ and children are ‘hideously underprepared’ (Anaesthetist, North East England, MSS2).

Child engagement could be facilitated by health professionals who emphasised empowering children and giving them choices. Empowerment can be time-consuming, but helps to address children’s fears:

We’re not going to restrain them, or do anything that’s against their will. And that’s just, I think, an acceptance that even if it’s an urgent case, just to give them a little bit of time and not force them, which sometimes, when they feel a little bit cornered or caged inevitably just leads to more resistance. (Anaesthetist, North West England, MSS4)

##### Caregivers’ attitudes to premedications

Caregivers’ engagement is a facilitator to the acceptability of premedications, due to the need for consent. Caregivers largely accept premedications and view them as beneficial for children, and for themselves:

Most parents are quite accepting of the fact because I could sense that it stems from their hope or belief that this will help them get through the operation for the child and obviously save them coming back. (Anaesthetist, South East England, MSS3)

Anaesthetists reported it was rare for caregivers to not want a premedication if clinically recommended. They also reported requests for premedication:

Caregivers are often the ones that feel that they might need…when we go through the options of how do you think they're gonnacope with this, they are often the ones that push for the pre-med actually. Particularly in the younger children. (Anaesthetist, North West England, MSS4)

Caregivers were similarly positive about premedications, emphasising a willingness to try anything that could make things easier for their child:

Yeah, I think a premedication would be best for him because he does get a bit upset quite easily about things. So, I think if he’s just calmed down a little bit, it would be better for him, I think. (Caregiver, PSP3)

However, caregivers can be a barrier to the administration of premedications. Firstly, a caregiver can be unwilling to give children extra medication, regardless of which premedication is offered. Secondly, the possibility of a delayed stay in hospital could be a deterrent:

We always warn that the anxious ones, ones we see in pre-op, that if they’re an afternoon list particularly, there is that possibility that the pre-med won’t have worn off in time to get them home as quickly. And some parents actually just say, “Well, I prefer not to have a pre-med then.” (Anaesthetist, North East England, MSS2)

Thirdly, caregivers who have had a negative experience in the past may want to avoid any premedication:

Sometimes they might have had a bad experience with the child having pre-med on the previous procedure, so when they come in, they don’t want their child to have a pre-med because of how they reacted to the pre-med last time. (Research nurse, South East England, MSS9)

Caregivers can also be disappointed in the effect of a premedication:

When you give their child some midazolam or clonidine or both, and they’re on the lighter end of sedated, the parents will then say, “Oh well, that didn’t work. It’s useless.” […] sometimes I think if they’re expecting a calm, sleepy, totally chill child, and they don’t get one, then how that makes them reflect on you. (Anaesthetist, Scotland, MSS1)

This can relate to their expectations, which need to be managed. As noted above, caregivers may also recognise the need for trade-offs (e.g. accepting delayed recovery or challenging behaviour if a premedication allows the child to successfully undergo surgery).

##### Health professionals’ attitudes to premedication

Premedications were seen to play a recognised and important role in facilitating GA and avoiding the need for restraint in some children. Nevertheless, as discussed above there can be trade-offs for caregivers and health professionals, and premedications can impact on recovery and other planned procedures. The use of one or more premedications can also be made more challenging due to practical issues around communication, timing of administration etc. Attributes of a particular premedication can also be both a virtue and a risk (for instance, sleepiness for very anxious, uncooperative children may be helpful for caregivers and health professionals, but can also result in a longer recovery time, which has associated challenges, as discussed above).

Anaesthetists displayed different attitudes towards the use of premedication more generally. For example:

I don’t like to use premedication if I can because it…I think it’s a pharmacological form of restraint […] as opposed to a physical form of restraint. (Anaesthetist, Scotland, MSS7)

I don’t mind pre-med in kids because if they ask for it, I think I’m for it […] I’m quite happy to give them pre-meds if they want them. (Anaesthetist, North East England, MSS8)

Attitudes towards premedication may reflect different priorities and concerns. For instance, clinicians may prioritise trying to manage anxiety in a non-pharmacological way:

One of the anaesthetists said to me, oh, you know, “we do very few” or “we do very few pre-meds” because he said, “I am the pre-med”. So, you know, he was a jovial man, he was great with the kids and often you might be booked in for pre-med and he took them round to not having one. (Research lead, North East England, PSS14)

Alternatively, a more positive attitude to premedication may reflect how this approach can make the experience of GA ‘easier’ for everyone involved, and the benefits associated with this.

##### Health professionals’ attitudes to evidence

Evidence from an RCT is not necessarily a facilitator. While the interviews indicated a personal willingness to change clinical practice, participants acknowledged other anaesthetists may be less likely to do so:

You know what doctors are like. They’re terrible. They don’t necessarily follow, A, they don’t necessarily follow guidance. B, they don’t necessarily follow the evidence base. They do what they think works and so they will, they will use whatever they have always used and sometimes they don’t want to change it. (Anaesthetist, Scotland, MSS7)

With any new option, individual take-up would likely be slow, starting with a few people and spreading. A key aspect was word-of-mouth accounts of success, from both nurses and anaesthetists. In addition to a trial, first-hand experience reported by trusted colleagues was important in terms of changing behaviour:

Word of mouth is important, I think, because we sort of, not everyone experiences everything, so someone you trust and work with has experience, let’s say, has administered melatonin and found the drug to be good. That would be something on top of my head the next time I needed to pre-medicate someone and that idea would instantly come to my mind if I have known that someone I trust has used it quite reliably. (Anaesthetist, South East England, MSS3)

##### Health professionals’ skills

A skilled health professional may facilitate the acceptability of any premedication. Anaesthetists described understanding and meeting the different needs of individual children. Practically, this involved observational skills, effective communication and building trust. While talking to a child, an anaesthetist might pick up clues about the child from their non-verbal behaviour:

You kind of get a sense of what they’re going to take in, what they’re going to understand, what they’re not going to understand. (Anaesthetist, Scotland, MSS1)

More generally, staff spoke about ensuring children were informed about what was happening (in a way appropriate to the individual child and his/her level of understanding):

I’ll explain to them, often will explain to the child what we’re planning to do and the reason behind it. […] And I will often try to be guided by the children as to their wants […] the communication with kids and the families is probably the most important. (Anaesthetist, Scotland, MSS5)

Staff also emphasised the importance of building and maintaining trust,

You’re having to really build up that trust again. So, whatever plan I’ve made with them in clinic, thatis what we do. We don’t deviate from that plan. We do exactly what we promised them, and we don’t change anything. (Anaesthetist, North East England, MSS2)

Overall, anaesthetists emphasised being child-led and involving the child in the process in order to achieve cooperation, a pleasant experience and the successful administration of anaesthetic.

Nurses also work to build relationships with children in order to help facilitate the process:

And so we had to be, you know, we had to make friends first so that we could just chat and play and start building up a relationship before we could sort of, you know, broach the subject of anything that was gonnahappen thereafter. (Research nurse, Yorkshire and Humber, MSS6)

Anaesthetists highlighted the importance of ‘friendly’ nurses, and that nurse/child interactions were key to achieving cooperation:

I’ve got a couple of nurses who, you know, they’re like a kid whisperer, like mid-50s. They’re like a granny and they’ll just come in and they’ll have a perfect rapport with kids. And it just makes your life so much easier. And in fact, that’s what, that makes a huge difference to those people being there probably more so to more children than any of the pre-meds that we’re using. (Anaesthetist, Scotland, MSS5)

In the MAGIC trial, children were accompanied by a research nurse which may have had an impact on anxiety:

I’ve found some of them when you first spoke to them might be anxious but by the time you’ve…before you’ve even given the pre-med, it feels like you’re already more relaxed anyway, just because you had that time to help calm them down a bit. (Research nurse, Yorkshire and Humber, PSS15)

Caregivers were also positive about the presence of a research nurse throughout the trial and commented on their ability to interact with children.

##### Managing known risks

Staff acknowledged the potential for children to reject premedication if administered orally as a recognised challenge. Spitting out raises issues as it is not clear how much has been swallowed and whether re-dosing is appropriate. An alternative tasteless option can be offered, but having spat out one premedication, children may not accept another. This can lead to various non-ideal situations: a potentially suboptimal anaesthetic without premedication, a delay to help the child calm down, or cancellation of the operation, with subsequent cost implications.

Recognising and managing this known risk can facilitate acceptability. Staff may prepare children for the taste, and outline ways to make the experience more pleasant:

I would say that if they have – explain that it tastes yucky like an antibiotic and explain that it’s a small amount of medicine but it’s going to help them get through this and that we’ll give them some juice or an ice lolly afterwards so they can have that to take the taste away. (Anaesthetist, Scotland, MSS5)

The taste of midazolam means administration might require additional work, particularly if children have had a prior negative experience.

Other children will accept midazolam if the experience is made more pleasant, for example, if the taste is masked or if they have already been given another premedication, such as clonidine. Outside of the MAGIC trial, a more expensive and palatable formulation of midazolam could be used (although not all agreed on palatability). When premedications are tasteless, administration can be achieved through concealment, which can be agreed at the pre-assessment (bringing a child’s usual cup to avoid using a syringe).

##### Working practices for premedication

Working practices include systems and processes for the procurement, selection and administration of premedication, and how practices work within a setting (this includes considerations of layout; for instance, how close are the ward and theatre and what is the impact of transfer time?). Working practices can impede or facilitate the use of any premedication. For instance, there can be difficulties within a particular setting relating to communication:

The biggest issue is between communicating things between theatre and the wards. It’s, yeah, you know, if you had a button you just press like to say, you know, give premedication now, that would be fine […] Because they’re too busy doing something else, then they don’t have time to give the premedication at the time that you think they’re giving it. (Anaesthetist, Scotland, MSS7)

Recognising the wider issues that practically impact on clinical care highlights challenges using premedications, as this same anaesthetist explained: ‘We’re fighting time as much as anything in order to try and maximise the efficacy of it.’. Such issues provide the context in which premedications are used and any new option for premedication is assessed.

Working practices also impact on potential departmental changes regarding premedication. Outside of the MAGIC trial, a particular premedication, such as midazolam, could be specified as the first-line option. This can reflect departmental consensus:

Making midazolam our first line is because I wrote it into the protocol. We discussed it, we agreed it, and it went into the protocol. And that’s what we do. (Anaesthetist, North East England, MSS2)

While individual anaesthetists are independent clinicians, they may therefore need to justify departing from departmental protocols. For a new premedication, processes could potentially be a facilitator or barrier; processes could be described as ‘easy’ (Anaesthetist, Scotland, MSS1), simply informing the pharmacy and colleagues that a new premedication was available, or ‘long’ (Anaesthetist, South East England, MSS3), involving discussing costs, form filling and additional training for nurses. If costs are significant, decisions may be taken out of the hands of clinicians.

## Discussion

MAGIC was a UK-based, double-blinded, multicentre parallel randomised controlled non-inferiority trial. The trial found melatonin was inferior to midazolam in terms of reducing anxiety, based on the primary outcome of pre-operative distress [15]. However, the acceptability of premedications related to factors other than efficacy at reducing pre-operative anxiety.

Within these interviews, anaesthetists viewed midazolam as largely effective. Overall, they suggested effectiveness can vary, particularly among children on the autistic spectrum. Nonresponse to midazolam has been reported elsewhere (e.g. 14.1% of 262 children) [16]. Among stakeholders and participants in MAGIC, melatonin was viewed as potentially effective, although some negative attitudes were reported among anaesthetists more widely (i.e. those not involved in MAGIC). However, the results of MAGIC demonstrated inferiority of melatonin when compared to midazolam as an anxiolytic premedication in children [15]. Within MAGIC, anaesthetists and nurses were assuming conclusions about the effectiveness of melatonin based on observations of children (although both were blinded, familiarity with midazolam meant they did have an idea which premedication had been administered). Based on a small sample, most caregivers felt their child had received an effective premedication.

Administering any premedication can be a challenge. While taste is relevant when providing an oral premedication, spitting out may reflect a lack of trust or higher levels of anxiety rather than the palatability of a particular premedication. In addition, recovery from GA following midazolam can be challenging for caregivers and theatre nurses who have to deal with a distressed child. There may also be behavioural impacts at home, in both the short and longer-term. When considering acceptability, it is important to consider context; caregivers may focus on the positives of the situation (the child has undergone necessary treatment and has woken up following GA). Challenges such as distressed behaviour, delayed discharge and post-operative symptoms at home may be ‘acceptable’ in this context. This highlights the importance of trade-offs for children, caregivers and health professionals, and raises questions about knowing what those trade-offs might be, versus more uncertainty.

Another issue post-recovery is the presence of an amnesic effect. While health professional participants noted the amnesic effect of midazolam can be a benefit [9], this also results in children who need to return for future treatment forgetting that they felt calm and were able to successfully undergo a surgical procedure. Melatonin may allow children to build up more positive associations over time (possibly alleviating the need for a premedication). Viewing children who are known to need future treatment as a subgroup may be helpful here, and more could be done to identify subgroups among anxious paediatric patients pre-GA.

Caregivers are potentially more likely to accept both melatonin and midazolam where there is relatively positive personal familiarity, whether as a premedication or in a different context. Similarly, anaesthetists made connections between familiarity and use, both for themselves and theatre nurses. While other research has explored children and parent experiences of GA [17, 18], these do not include experience of premedication. More generally, previous experience of GA within a family may lead to acceptance of GA for a subsequent child, but does not necessarily reduce parents’ emotions and experiences of fear [17]. For clinicians, the idea that an alternative premedication would need to be ‘massively more efficacious’ (as one anaesthetist put it) to be adopted is unrealistic in a non-inferiority trial; it may be that a desire to continue to use a familiar approach leads to unrealistic hypothetical conditions being applied to a new alternative, and this is important for those designing trials to recognise.

MAGIC compared melatonin against midazolam as the ‘current standard premedication given to an anxious child ahead of surgery’ [11]. However, other premedications are available and may be the standard first line within some departments, whether used alone or in combination with midazolam (such as clonidine and dexmedetomidine). Thus individual anaesthetists may already use alternatives to midazolam that address some of the recognised issues (for example, being more palatable to children), and are therefore clinically more acceptable. The lack of equipoise was a barrier to recruitment to MAGIC, as some anaesthetists want to use a familiar premedication or combination of premedications [12], and this is a recognised barrier to trial recruitment [19].

Some children have previous experience of a surgical procedure under GA, and anaesthetists will draw on these experiences when deciding how to address the child’s anxiety. If a particular approach has been successful in the past, an anaesthetist may be unwilling to try something different [12]. As we have demonstrated, predictability is important for anaesthetists, and alternatives to midazolam and melatonin may be preferred if effective for a particular child. In the context of a non-inferiority trial, it is important to recognise that MAGIC was not aiming to demonstrate melatonin as more efficacious. Had MAGIC shown melatonin to be non-inferior to midazolam with fewer side effects, health professionals would have faced trade-offs in their decision-making; however, midazolam may still have been preferred. Shared healthcare decision-making appears important, with the decision on the appropriate premedication being based on individual patient trade-offs, as well as clinician preferences and trade-offs. Exploring the attributes of premedications that matter to children, caregivers and clinicians is important to understand exactly what those trade-offs are, as well as to inform the feasibility of future trials on the effectiveness of new premedications. Like all shared health care decision-making, it is essential clinicians fully explain all factors to the patient/caregiver to enhance discussions on treatment selection.

Health professionals’ attitudes to evidence is another important factor. While doctors are generally in favour of evidence-based medicine [20], health professional participants emphasised the importance of word-of-mouth reports, in this case, hypothetical examples of success with melatonin from trusted colleagues, to encourage changes in practice. This issue has been identified elsewhere in relation to induction; ‘despite numerous arguments to rationalise the choice of a drug for induction of anaesthesia, most of the time the use of a specific drug is guided by the habits of anaesthetists’ [21]. More generally, the interviews with health professionals indicated how slow individual take-up could contribute to a time lag in the translation process [22].

Overall, understanding the potential acceptability of two options for paediatric premedication pre-GA outwith the context of a trial involves recognising the wider context in which these are prescribed, for children, caregivers and health professionals. This includes considering factors such as attitudes, abilities and experiences of different individuals involved, and other available options for premedication and working practices within individual settings. While these interviews were limited on their ability to *fully* explore the multifactorial decision-making processes involved in prescribing premedications for children pre-GA, this study provides a starting point to further understand the context and inform future trials.

### Strengths and limitations

This paper provides a detailed overview of perceptions of child, caregiver and health professional acceptability of premedications provided prior to GA, and specifically the acceptability of midazolam and melatonin. By drawing on semi-structured interviews that focused on the topic of acceptability, the research team were able to explore this issue and take account of multiple perspectives. To our knowledge, no previous studies have used a qualitative approach to explore the use of premedications for paediatric patients prior to GA. This paper develops our understanding of the context of premedication use among paediatric patients and highlights important considerations for trial design in this area.

There were several limitations. Firstly, very few caregivers agreed to be contacted, and of those that did, several did not respond to attempts to contact. Due to the time involved in getting caregivers’ details to the qualitative research team, recall may have been affected, and caregivers and children often did not have much to say about the topic. It was difficult to arrange to speak to children; some caregivers felt they would not respond well to being interviewed. In addition, there were different dynamics when children and caregivers were interviewed together (and caregivers commented on what children said) and when caregivers were present while children spoke to the interviewer by telephone. Caregivers may have also responded differently to questions depending on whether a child was present or not. Although some children, particularly the youngest, may have felt more comfortable with a caregiver taking a more active role, it is important to recognise that different interview dynamics can impact on research findings. Children also reported not remembering what happened during MAGIC, and as midazolam has an amnesic effect, this may be a factor. Additionally, asking children about an experience that made them anxious may be difficult for them, and was challenging for the researcher, particularly when there was little opportunity to build rapport. Interviews for the main trial were conducted in December 2020 and January 2021, when restrictions related to COVID-19 were in place. Pressures related to the pandemic may have contributed to interviews about MAGIC being given low priority.

Secondly, although interviews with caregivers/children who refused to take part in MAGIC would have been useful, this was not possible, due to the challenges of sharing contact details of patients outside of the trial. In terms of acceptability, it is important to consider the views of those unwilling to take part in this trial, who may view melatonin as an unacceptable option (or who may be unwilling for their child to participate in any trial). As all interview participants have consented to their children receiving midazolam or melatonin, both are acceptable to some degree.

Thirdly, further interviews with other health professionals would have been beneficial, in particular anaesthetists with differing levels of experience (including trainees) and nurses working in surgical day units. However, due to pressures related to COVID-19, anaesthetists were not always able to focus on research. Although PIs were asked to inform colleagues about the interviews, no PI responded to indicate they had done this, and the research team did not receive any expressions of interest.

### Areas for future research

Outside of a specific RCT, there is scope for further research to explore the perspectives of children, caregivers and health professionals regarding the use of premedications to address anxiety prior to surgical or medical procedures under GA. This research could purposively sample children and caregivers who reject any premedication, and anaesthetists who manage anxiety in other ways, in order to gain a wider insight. The framework outlined above could inform future interviews. There is also scope for further exploring the acceptability of premedications at different time points using multiple interviews and the Theoretical Framework of Acceptability [7].

Whilst midazolam is accepted as the standard premedication for children in the UK, and the MAGIC trial identified melatonin was inferior to midazolam, there remains a need for a premedication with a better side effects profile. However, this study has identified that decision-making on premedication choice in children is multifactorial. Future RCTs in this area are likely to be non-inferiority design, with midazolam being the premedication to match in effectiveness. Therefore, a study such as a discrete choice experiment could explore the attributes of premedication important to children, caregivers and clinicians to ensure the proposed experimental treatment is acceptable within this population and to prescribing clinicians. This study could also identify where a new proposed premedication may best fit within the population i.e. subgroups where it may be best (or worst) placed.

One solution to balancing the complex choices being made when selecting a premedication is the development of a multi-dimensional evaluation index, which in addition to effectiveness and side effects considers other dimensions such as administration experience, recovery profile, prior patient experiences and costs. Future work should therefore aim to quantify all important dimensions through formal benefit-risk methods to understand the importance of weightings which would be assigned to each dimension and the trade-offs between them [23]. To enhance the precision and personalization of drug selection a tool available to clinicians and patients could be created to help assess personalised trade-off (see the Fracture Risk Assessment Tool as an example) [24].

## Conclusions

Future clinical trials evaluating the effectiveness of premedications in children prior to general anaesthesia need to consider that such choices are multifactorial. The MAGIC study found melatonin was less effective at reducing anxiety (pre-operative distress) when compared with the standard of care, midazolam. However, this study identified a range of barriers and facilitators that may impact on the acceptability of an individual premedication, and on the use of premedications more generally. For example, some anaesthetists favour premedications which induce drowsiness, others avoid premedications for the same reason (or may do so in certain situations).

While children, caregivers and health professionals are open to alternatives to midazolam, this is likely to vary by subgroup and will involve trade-offs in terms of benefits. There remains a need for a premedication with a better side effects profile to midazolam. Further RCTs in this area could consider discrete choice experiments for children, caregivers and health professionals around these trade-offs regarding effective premedications.

## Data Availability

The datasets analysed during the current study are available from the corresponding author on reasonable request.

## Abbreviations

CI: Chief investigator
ENT: Ear, nose and throat
GA: General anaesthesia
GCP: Good Clinical Practice
IMP: Investigational Medicinal Product
IV: Intravenous
MAGIC: Melatonin for Anxiety prior to General anaesthesia In Children
mYPAS: Modified Yale Preoperative Anxiety Scale
NHS: National Health Service
PI: Principal investigator
RCT: Randomised control trial
